# Age and Gender Effects on Genotoxicity in Diesel Exhaust Particles Exposed C57BL/6 Mice

**DOI:** 10.3390/biom11030374

**Published:** 2021-03-02

**Authors:** Joong Won Lee, Jin Sik Kim, Hee Jae Lee, Ji-Hye Jang, Ja-Hyun Kim, Woo Jong Sim, Yong-beom Lim, Ji-Won Jung, Hyun Joung Lim

**Affiliations:** 1 Department of Chronic Disease Convergence Research, Division of Allergy and Respiratory Disease Research, Korea National Institute of Health, Chungju 28159, Korea; won3@korea.kr (J.W.L.); heejaewith@korea.kr (H.J.L.); jikong0214@korea.kr (J.-H.J.); 2 GLP Center 1, Korea Conformity Laboratories, Bio Division, Incheon 21999, Korea; bioman@kcl.re.kr (J.S.K.); Jahkim@kcl.re.kr (J.-H.K.); kjsim@kcl.re.kr (W.J.S.); 3 Department of Materials Science and Engineering, Yonsei University, Seoul 03722, Korea; yblim@yonsei.ac.kr

**Keywords:** fine particulate matter, diesel exhaust particles, genotoxicity, micronucleus assay, comet assay, aged, effect modification

## Abstract

There is growing evidence that the accumulation of DNA damage induced by fine particulate matter (PM_2.5_) exposure is an underlying mechanism of pulmonary disease onset and progression. However, there is a lack of experimental evidence on whether common factors (age, gender) affect PM_2.5_ induced genomic damage. Here, we assessed the DNA damage potency of PM_2.5_ using conventional genotoxicity testing in old male and female mice aged 8 and 40 weeks. Mice were intratracheally instilled with diesel exhaust PM_2.5_ (DEP, NIST SRM 1650b), twice a week for 4 weeks. Exposure to DEP was not associated with an increase in the frequency of micronucleated polychromatic erythrocytes and did not induce a systemic genotoxic effect in the bone marrow. Meanwhile, the results from the comet assay showed a significant increase in DNA damage in DEP exposed mouse lung specimens. The positive relationship between DEP exposure and DNA damage is stronger in the older than in the younger group. Statistical analysis showed that there was a modifying effect of age on the association between PM_2.5_ exposure and DNA damage. Our results suggest that the age factor should be considered to better understand the cellular adverse effects of PM_2.5_.

## 1. Introduction

Small particles less than 2.5 micrometers or less in diameter are defined as fine particles (PM_2.5_) and have the greatest health risk [1]. PM_2.5_ poses the longest residence time in the atmosphere and the easiest and deepest penetration into the alveoli and bloodstream due to their small size and large surface area, which implies more toxicity [2]. Fine particles emitted during the combustion process of diesel engines occupy a high proportion of urban PM_2.5_ generation sources [3,4]. Diesel Emissions are known to be a pulmonary carcinogen, based on sufficient evidence that exposure is associated with an increased risk for lung cancer [5]. In several experimental studies, increased inflammatory responses, cellular senescence, oxidative stress, and DNA damage induced by diesel exhaust PM_2.5_ (DEP) exposure have been demonstrated as plausible pathways for the onset and progression of lung disease [6,7,8].

The DEP used in this study was NIST SRM 1650b which is composed of polycyclic aromatic hydrocarbons (PAHs), nitro-PAHS, organic and elemental carbon, and reactive metal [9,10]. NIST SRM 1650b’s characterized mean particle diameter is 0.18 μm with a strong peak in the ultrafine range making it ideal in investigating the carcinogenic and genotoxic potential of fine particles. Results from cell culture experimental models indicate that NIST 1650 elevated the cellular level of DNA damage in human alveolar carcinoma and monocytes [11,12,13]. Nose-only inhalation exposure of mice to NIST 1650 increased 8-oxodG in lung tissue [14]. Another inhalation study found the application of NIST 1650 to female 8-week-old BALB/CJ mice led to DNA strand breaks in broncho-alveolar lavage (BAL) cells and/or lung tissue [12]. Guinea pigs (aged 40–50 days) exposed by intratracheal instillation to NIST 1650 had increased levels of oxidative DNA damage in the lung [15]. In addition, oral administration of NIST 1650 increased the level of bulky DNA adducts in the lung of male 8- and 9-week-old rats [16,17]. As such, the current literature suggests that NIST 1650 could potentially induce genotoxicity through direct or indirect mechanisms. However, as mentioned above, most studies have been done using young adult animals; therefore, information about the genotoxic potential of DEP in older mice is limited.

It is acknowledged that age and gender are important biological parameters that now are required to be provided in study designs, analyses, and reporting in experimental animal and human studies [18,19]. However, only a few biological monitoring studies have been reported showing that age and sex can influence DNA damage from exposure to environmental pollutants [20,21]. Heuser et al have shown that adult females are the principal population affected by air pollutants from vehicle emissions [20]. In a study comparing the level of damaged cells in adult, sub-adult, and juvenile rodents to identify possible relationships between age and DNA impairment, increases in the level of damaged cells associated with age were found for wild rodents in coal regions [21]. In a study assessing micronucleus formation in rats after chronic exposure to DEP, no sex-based differences in MN frequency were observed [22]. The current understanding of the common variables (age, gender) effect of PM_2.5_ induced DNA damage is unclear and limited. Therefore, in the present study, we compared the difference genomic damage by intratracheal instillation of DEP in male and female 8- and 40-weeks-old male and female mice.

## 2. Materials and Methods

### 2.1. Characterization of the Prepared DEP

DEP (SRM 1650b; National Institute of Standards and Technology, Gaithersburg, MD, USA) were purchased from Sigma Aldrich and dispersed in ultrapure water. The prepared DEP has been physically and chemically characterized to determine its impact on the tested biological cells. The DEP sample’s particle size was determined by Dynamic light scattering (DLS). Briefly, the fluctuation of the scattered light intensity, which is caused by the Brownian motion of the particles in suspension, is measured over time. DLS experiment was performed at 25 °C with ELS-1000ZS (Otsuka Electronics, Osaka, Japan). The polydispersity index (PDI), which indicates the quality concerning the stability and the extent of uniformity and homogeneity of the particle emulsions, was also determined. The surface charge of DEP was measured in distilled water (DW) using the same equipment. The pH of the DEP solution was measured with a benchtop pH meter (Fisher Scientific, Waltham, MA, USA) to evaluate the compatibility of the sample to the biological experiments. The surface structure and the chemical composition of the prepared DEP were investigated by the scanning electron microscope with an energy dispersive X-ray (EDX) spectrometer (EMAX 3.0, Horiba, Kyoto, Japan). The sample was fixed to the stub and Pt-coated. EDX spectrum of DEP was collected at accelerating voltage 15 keV and working distance of 14.9 mm.

### 2.2. Animals

C57BL/6J (8 weeks and 40 weeks of age) mice were obtained from Animal Facility of Aging Science, the Korea Basic Science Institute (Gwangju, Korea). Young and aged mice were housed in a temperature and humidity-controlled room (22 °C ± 3 °C, 50% ± 10% humidity) with 12 h light: 12 h dark cycle. Mice were allowed access freely to standard laboratory chow and tap water. The study protocol with animals was approved by the Institutional Animal Care and Use Committee of the Korea Institute of Toxicology (Approved protocol number: 2005-0139, 0140).

### 2.3. Intratracheal Instillation of DEP

**Figure 1:**
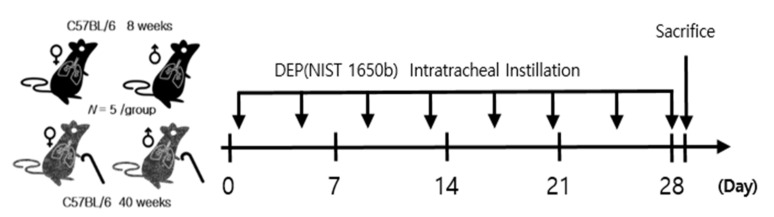
Schematic illustration of the experimental design. Mice were intratracheally instilled with DEP (diesel exhaust particle). The control group was treated with 50 µL ultrapure water. (n = 5 per group).

**Figure 2:**
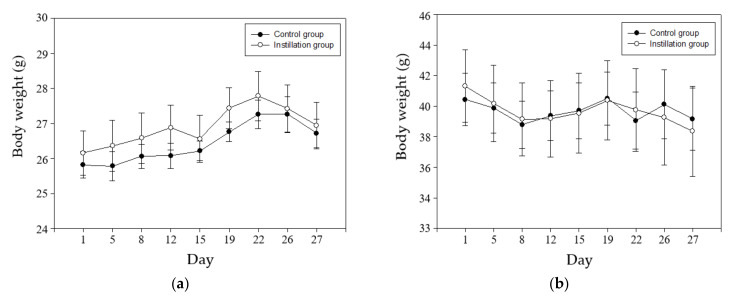

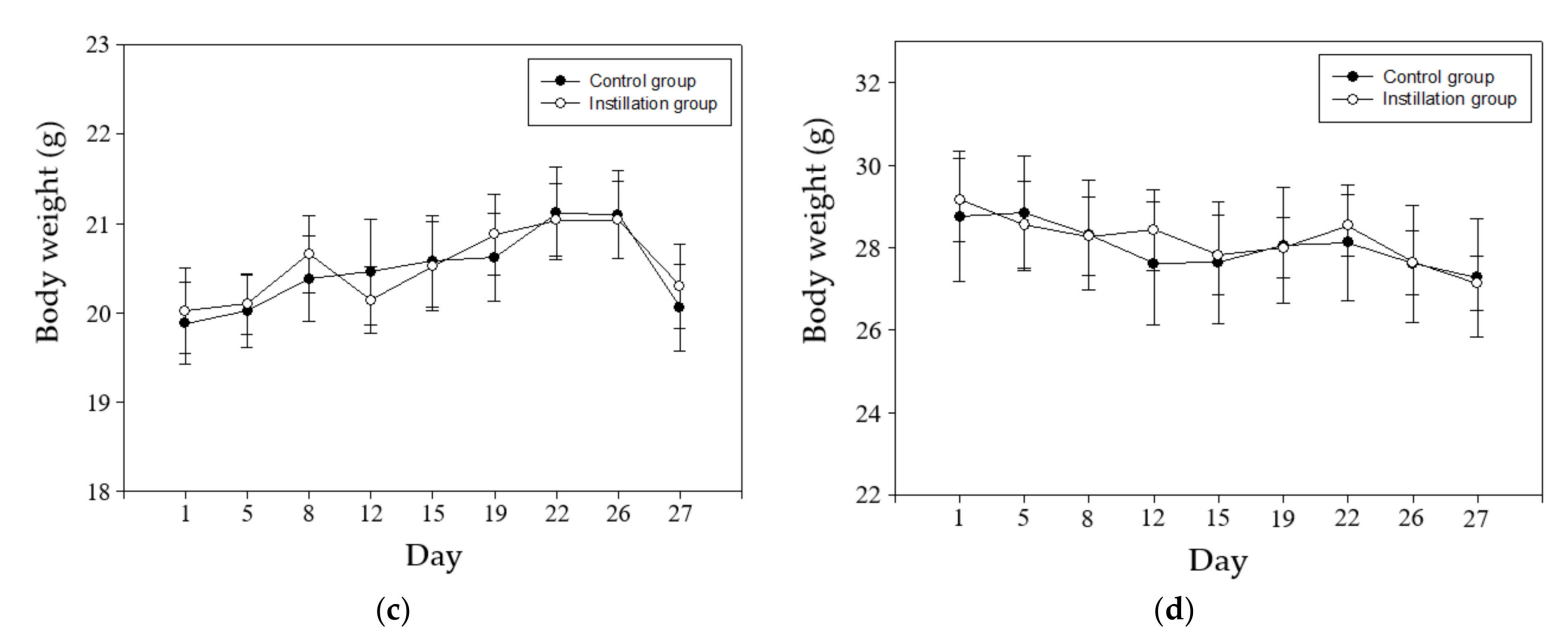
Body weight (mean ± SE) change in vehicle (control) or Diesel Exhaust Particle (DEP) instilled in mice. (**a**) Young male mice (8-week-old); (**b**) Old male mice (40-week-old); (**c**) Young female mice (8-week-old); (**d**) Old female mice (40-week-old).

After five days of adaptation to the laboratory conditions, mice were placed in experimental groups. Under isoflurane anesthesia, each mouse was intratracheally instilled with vehicle or 50 µg of diesel PM (Standard Reference Material-1650b; National Institute of Standards and Technology, Gaithersburg, MD, USA) dispersed in 50 µL ultrapure water. DEP instillation dose was referred to previous studies [23,24,25,26,27,28,29]. Our collaborators have shown that 50 and 100 µg of DEP exposure (three repeated intratracheal instillation in a week) induced lung inflammatory reactions in mice [27]. In this study, the tested dose has been determined as the lowest one able to induce lung inflammatory response in DEP exposed mice. Intratracheal instillation was performed twice a week for four weeks (Figure 1). After 4 weeks of instillation, mice were sacrificed with isoflurane inhalation. Bodyweight measurement was performed before exposure. As a result, there was no significant difference in body weight change between DEP-exposed and unexposed control in both genders (Figure 2).

### 2.4. In Vivo Micronuclei Assay

Bone marrow cells were obtained from the femurs immediately following sacrifice and the bone marrow was collected in a 1.5 mL microcentrifuge tube containing 1 mL of fetal calf serum and centrifuged 5 min at 1000 rpm. Two air-dried smears were prepared before fixation with methanol and staining with an acridine orange solution. A drop of 0.04 mM acridine orange solution dissolved in phosphate buffer was placed over the fixed cells and covered with a coverslip. Micronuclei (MNs) were evaluated as described in previous publications [23]. The number of micronucleated polychromatic erythrocytes (MN-PCEs) among 4000 polychromatic erythrocytes (PCEs) per animal was examined within a day using a fluorescent microscope, and coded slides were scored blindly by one expert scorer. With fluorescence staining, normal colored red blood cells (NCE) become opaque. Therefore, one slide per animal was stained with May–Grünwald and Giemsa solutions. To assess bone marrow toxicity, the PCE/(PCE + NCE) ratio was calculated based on a total of 500 erythrocytes in these stained slides.

### 2.5. In Vivo Comet Assay (Single Cell Gel Electrophoresis Assay)

Lungs were obtained from five male and five female mice in each test group, respectively. The lungs were minced and suspended in chilled PBS, and gently homogenized in ice using a tissue grinder (Kontes, Vineland, NJ, USA). The cell suspensions were then transferred to a nylon cell strainer (BD Falcon, Franklin Lakes, NJ, USA) in sterile tubes. The viable cell counts for the cell suspensions were determined using the trypan blue dye exclusion method. For the first layer, 1.0% normal-melting agarose was dropped onto a frosted microscope slide. The cell resuspensions (2 × 10^4^/10 µL) were then mixed with 85 mL of 0.7% low melting agarose and rapidly spread on the first layer. Finally, 85 mL of 0.7% low-melting agarose was used as the top layer. The prepared slides were then soaked in an alkaline lysing solution (2.5M NaCl, 100 mM Na2-EDTA, 10 mM Tris-HCl, 1% Triton X-100, and 10% DMSO, pH 10.0) for 1 h at 4 °C. Thereafter, the slides were washed in distilled water for 10 min, placed in a horizontal electrophoresis chamber, and electrophoresed in an alkaline buffer (1 mM Na2-EDTA, 300 mM NaOH, pH 13) for 25 min at 20 V and 275 mA. Next, the slides were gently washed in a neutralization buffer (0.4 M Tris-HCl, pH 7.5) and immersed in 100% ethanol for 1 h. The slides were then washed in a neutralization buffer (0.4 M Tris-HCl, pH 7.4) and immersed in 100% ethanol for 1 h. Finally, slides were stained with 100 μL of SYBR Green solution, and images of 100 randomly selected cells were analyzed from each animal using a Comet Assay IV analysis system (Instem-Perceptive Instruments Ltd., Suffolk, Halstead, UK).

### 2.6. Statistical Analysis

Statistical analyses were performed using SPSS 12.1 (Chicago, IL, USA), and the data expressed as the mean ± standard error (S.E.). A one-way analysis of variance (ANOVA) and T-test were also applied to test all the data. The effect modification was confirmed using multiple linear regression model analysis. A value of *p* < 0.05 indicated statistical significance. 

## 3. Results

### 3.1. Physical and Chemical Characteristics of DEP

**Figure 3:**
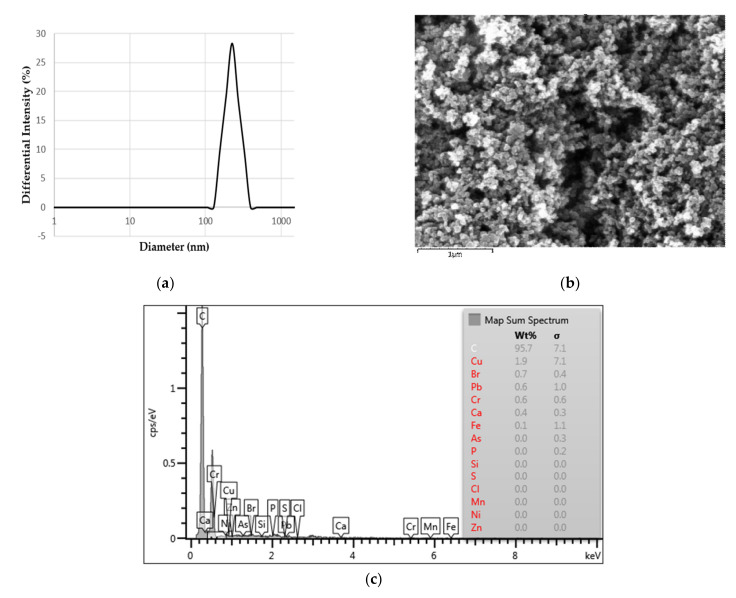
Physical and chemical information about the DEP (diesel exhaust particle). (**a**) The size distribution of DEP in dH_2_O; (**b**) SEM image of DEP; (**c**) EDX (energy-dispersive X-ray) spectrum of DEP.

DEP dispersed in DW was analyzed to identify their physiochemical properties. The pH of dispersed DEP appears to be neutral (Table 1.) which is in a range of physiological conditions. The average size of DEP in DW was 268.7 nm (Table 1), which was confirmed to be PM_2.5_. The PDI value of DEP samples was 0.143 which indicates a narrow size distribution and homogeneous distribution of DEP [30]. Zeta potential of DEP in DW was found to be −26.29 mV. This value indicates sufficient repulsion force between the particles to attain good colloidal stability in the suspension. The SEM image of the DEP is illustrated in Figure 3b. It emphasizes that the particle surface is spherical. EDX spectrum in Figure 3c indicates that more than 95 wt% of DEP is carbon. With EDX analysis, elements in DEP include copper, bromine, lead, chromium, calcium, and iron.

### 3.2. Effects of DEP Exposure on Cytogenetic Damage

Micronuclei test based on OECD guideline 474 was performed to determine genetic damage to bone marrow cells caused by instillation exposure of DEP [31]. As a result of micronuclei assay in male mice, the frequency of micronuclei was 0.26 ± 0.03, 0.22 ± 0.04, 0.17 ± 0.03, and 0.16 ± 0.05 for young mice (8-week-old) control, 50 µg DEP- instilled young mice (8-week-old), old mice (40-week-old) control, and 50 µg DEP- instilled old mice (40-week-old) (Table 2). In female mice, the frequency of micronuclei were 0.15 ± 0.02, 0.11 ± 0.01, 0.10 ± 0.01, and 0.09 ± 0.01 for young mice (8-week-old) control, 50 µg DEP- instilled young mice (8-week-old), old mice (40-week-old) control, and 50 µg DEP- instilled old mice (40-week-old) (Table 3). In male and female mice, DEP instillation did not induce the DNA damage of mice bone marrow cells. DEP did not cause a statistically significant increase in the frequency of micronuclei when compared with young mice (8-week-old) control. In addition, the PCEs ratio of 500 erythrocytes (PCE/(PCE+NCE)), as an index of cytotoxicity, did not significantly decrease in PCE/(PCE+NCE) ratio compared to young mice (8-week-old) control in both genders. These results indicate that DEP had no systemic cyto/genotoxic effect in the bone marrow.

### 3.3. Effects of DEP Exposure on DNA Damage

**Figure 4:**
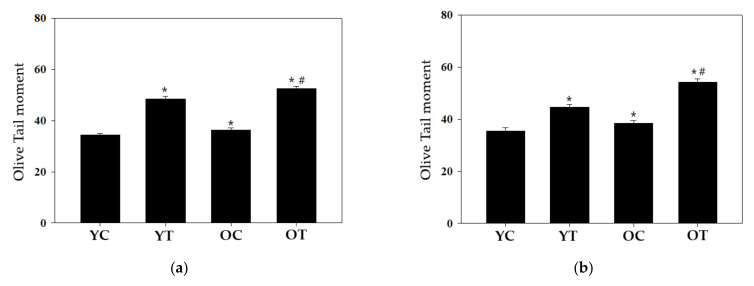
Quantitative analysis of C57BL/6 lung-cell DNA damage after DEP exposure using single-cell gel electrophoresis (comet assay). (**a**) Male mice; (**b**) Female mice. YC: young mice (8-week-old) control, YT: 50 µg DEP- instilled young mice (8-week-old), OC: old mice (40-week-old) control, OT: 50 µg DEP- instilled old mice (40-week-old). * *p* < 0.05 when compared with the young mice control. # *p* < 0.05 when compared with the old mice control.

It is well-known that Comet assay is a very useful DNA damage detection technique for eukaryotic cells [32,33]. Thus, Single cell gel electrophoresis was performed to investigate the DNA damage effect of the DEP. The comet assay was conducted on the C57BL/6 mice lung cells. We used Olive tail moment (OTM) as the parameter that provides a good correlation with the dose of the genotoxic agent in a Comet assay [34]. OTM expressed in arbitrary units, is defined as the product of the tail length and the fraction of total DNA in the tail. As a result of the comet assay in male mice, the OTM value was 34.36 ± 0.63, 48.40 ± 1.06, 36.25 ± 0.99, and 52.52 ± 0.85 for young mice (8-week-old) control, 50 µg DEP-instilled young mice (8-week-old), old mice (40-week-old) control, and 50 µg DEP-instilled old mice (40-week-old) (Figure 4a). DEP instillation enhanced the OTM value to a statistically significant level when compared with the young mice control (*p* < 0.05). In addition, Statistical significance was observed in the comparison between old male mice control and 50 µg DEP-instilled old male mice. According to female mice result, the OTM value was 35.52 ± 1.26, 44.64 ± 1.13, 38.38 ± 1.07, and 54.30 ± 1.20 for young mice (8-week-old) control, 50 µg DEP-instilled young mice (8-week-old), old mice (40-week-old) control, and 50 µg DEP-instilled old mice (40-week-old) (Figure 4b). In addition, Statistical significance was observed in the comparison between old female mice control and 50 µg DEP-instilled old female mice. These results indicate local genotoxic effects of DEP in mouse lung.

### 3.4. Effects of Age and Gender on DNA Damage in DEP-Exposed Mouse Lung

Multiple linear regression analysis using age, gender, DEP exposure as the independent variable was performed to investigate the association between DEP exposure and DNA damage, as well as between individual factors (age or gender) and OTM value from comet assay. Table 4 shows the significant association between DEP exposure and OTM value (β = 13.84, 95% CI = 12.39–15.28), as well as between Age and DNA damage (β = 4.63, 95% CI = 3.18–6.07), but gender did not show a significant association with DNA damage (β = 0.32, 95% CI = −1.12–1.77) (Table 4).

**Figure 5:**
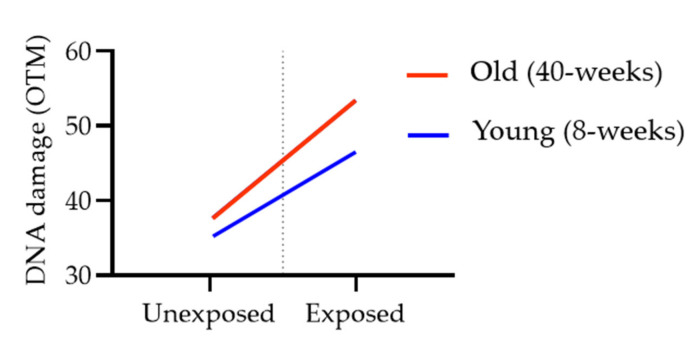
Interaction plot for DEP exposure × Age.

Stratified analysis was additionally performed to determine whether age is an effect modifier of the association between DEP exposure and DNA damage. Statistically, significant associations were observed between DEP exposure and DNA damage in both young and old age. There was a greater increase in DNA damage with DEP exposure in the older group (β = 16.02) than in the younger group (β = 11.58). In the interaction plot, we see a difference among the older age category compared to the younger age category indicating the presence of interaction (Figure 5). Furthermore, the interaction term (DEP exposure ∗ Age) is statistically significant (*p* < 0.0022). These statistical analysis results suggest the presence of effect modification of age on the relationship between DEP exposure and DNA damage.

## 4. Discussion

The fine particle matters used in this study were standard reference material generated by diesel engine emissions (NIST 1650b). This material has been well chemical characterized for several PAHs, nitro-PAHs, elemental carbon, organic compounds, sulfates, nitrates, and trace amounts of metals and other elements [9,10] supporting toxicological research of these inherent compounds. Some of the characterized PAH found in NIST 1650b are known to be genotoxicity and mutagenicity including benzo[a]pyrene [35]. The average of the diameters of NIST 1650b is 0.18 μm which makes it feasible to investigate the potential health risk of fine range DEP. In this study, we dispersed DEP in ultrapure water and physicochemical properties of aqueous DEP were analyzed by DLS and SEM-EDX. The average size of prepared DEP in water was 0.26 μm, as determined by DLS, indicating that prepared DEP is slightly aggregated together in the aqueous environment (Table 1). However, the size of the DEP aggregates is still in the fine particle range, which is small enough for penetrating the lung barrier and enter circulation. Zeta potential of DEP in DW was slightly smaller than 30 mV or less than −30 mV which are considered better stability of a colloidal suspension [36]. It was considered that our samples remain approximately stable in the dispersed state. The shape of the DEP was also analyzed by SEM, which revealed that the particle surface is spherical (Figure 3).

In our animal model, the treatment concentration and method for instilling DEP into the lungs of mice were based on our collaborators’ previous publications that demonstrated that re-suspended NIST SRM 2975 DEP induces inflammatory reactions and ER stress in the lungs [27,28,29]. We exposed NIST 1650b at a concentration of 50 µg in DW to mice through the intra-airway instillation method. Body weights of mice remained constant during the experimental period, and no differences were observed among groups (Figure 2)

One of the aims of this study was to evaluate the genotoxic potential of a well-characterized standard DEP in mice through conventional genotoxicity testing. To evaluate the systematic genotoxicity in bone marrow, a micronuclei test based on OECD guideline 474 was performed. A single-cell gel electrophoresis assay (Comet assay) was conducted to detect the DNA damage in DEP exposed lung cells. In our animal model, micronucleus analysis could not confirm the difference between DEP exposed and unexposed control group (Table 2 and Table 3), indicating that DEP had no systemic cyto/genotoxic effect in the bone marrow. Because the target organ was the lung in our experiments, a sufficient concentration of DEP or its metabolites might not reach the bone marrow to induce micronuclei. Although, there is no previous systemic genotoxicity assessment for the reference DEP substance (NIST 1650) in animal models. Limited in vivo studies have investigated micronucleus formation in the bone marrow and peripheral blood cells after inhalation exposure to DEP, most of these studies have been reported negative results similar to ours. No increase in micronucleus frequency was found in the bone marrow of mice, rats, and hamsters after long-term (from 1 month up to 2 years) inhalation exposure to DEP [37,38,39,40,41]. One previous study has shown an increase in the number of micronuclei in polychromatic erythrocytes from hamsters inhaled DEP for 6 months [42]. These conflict results might be partly due to differences in the concentration and duration of exposure and detailed type of DEP. 

In this study, the intracellular DNA damage levels were also measured to determine the cause of local genotoxic effects following NIST 1650 exposure using the comet assay. The OTM was significantly higher in both the male and female mice lung directly exposed to DEP (Figure 4). Our Comet assay result is in line with the consistent results from several previous studies that have shown DNA damage responses after inhalation or intratracheal administration. Dybdahl et al. identified oxidative DNA damage and DNA adducts in the mice lung after DEP exposure [12]. Nose-only inhalation exposure of mice to NIST 1650 increased oxidative DNA damage in lung tissue [14]. Guinea pigs exposed by intratracheal instillation to NIST 1650 had increased levels of DNA damage in the lung [15]. In the lung treated with inhalation of DEP for 12 weeks increase the mutant frequency in transgenic rats and mice [43,44]. However, most studies in this field have been done using young adult animals; therefore, information about the genotoxic potential of DEP in older mice is limited. Therefore, we exposed groups of young adult (8 weeks old) and aged (40 weeks old) C57BL/6 mice to DEP by intratracheal instillation. Our present study has shown a significant increase in DNA damage in mouse lung exposed to DEP in both the young adult and aged groups (Figure 4). It has been well described that oxidative stress and inflammation as key steps in the process leading to DNA damage [45,46]. To date, reactive oxygen species (ROS) generation and oxidative stress induced by DEP are frequently reported [47,48]. Chronic DEP exposure induces inflammatory conditions in mice [24,49], subsequently inflammatory and epithelial cells release ROS which gives rise to DNA damage [46]. Considering that oxidative stress and inflammation response after DEP exposure can be somewhat attributed to our positive results of the comet assay.

We conducted a statistical analysis to determine how OTM values as dependent variables were affected by age, gender, and exposure of DEP as dichotomous independent variables. Multiple linear regression model analysis has shown a significant positive association between DEP exposure and DNA damage, as well as between age and DNA damage, but gender did not show a significant association with OTM value (Table 4). A stratified analysis provides a way to identify effect modification [50]. When there is effect modification, the measures of association in the subgroups differ from one another. In our stratified analysis, the OTM value was increased more with DEP exposure among older. We could also assess the presence or absence of the interaction by examining the association graphically (Figure 5). From these results, we would conclude that difference in the DNA damage outcome differs according to age and there is evidence of effect modification of age on the DEP-induced DNA damage in mice lung. It can be speculated that this interaction between age and DEP-induced DNA damage is due to age-related changes such as decreased DNA repair capacity, epigenetic shifts, and altered RNA and protein profiles of xenobiotic metabolic pathways [51,52,53].

## 5. Conclusions

In our animal model, DEP exposure was associated with a significant increase in DNA damage in mouse lungs indicating the local genotoxic potential of DEP. In addition, to the best of our knowledge, we provide for the first time experimental evidence demonstrating the modification effect of age on genotoxicity in mice exposed to well-characterized DEP reference materials. Age should be one of the important independent variables in determining toxic reactions caused by fine particles.

## Figures and Tables

**Table 1:** Characteristics of the prepared fine particles.

Particle	Media	pH	Diameter (nm)	PDI	Zeta Potential (mV)
DEP (NIST 1650b)	dH_2_O	6	268.7	0.143	−26.29

**Table 2:** The frequency of micronuclei and PCE/(PCE+NCE) ratio in the bone marrow of male mice.

Groups	Animal No.	The Frequency of Micronuclei (%, Mean ± S.E.)	PCE/(PCE+NCE) (%, Mean ± S.E.)
Young mice control (8 weeks old)	5	0.26 ± 0.03	0.55 ± 0.01
DEP-instilled young mice (8 weeks old)	5	0.22 ± 0.04	0.55 ± 0.02
Old mice control (40 weeks old)	5	0.17 ± 0.03	0.54 ± 0.01
DEP-instilled old mice (40 weeks old)	5	0.16 ± 0.05	0.53 ± 0.01

**Table 3:** The frequency of micronuclei and PCE/(PCE+NCE) ratio in the bone marrow of female mice.

Groups	Animal No.	The Frequency of Micronuclei (%, Mean ± S.E.)	PCE/(PCE+NCE) (%, Mean ± S.E.)
Young mice control (8 weeks old)	5	0.15 ± 0.02	0.55 ± 0.01
DEP-instilled young mice (8 weeks old)	5	0.11 ± 0.01	0.55 ± 0.02
Old mice control (40 weeks old)	5	0.10 ± 0.01	0.54 ± 0.01
DEP-instilled old mice (40 weeks old)	5	0.09 ± 0.01	0.53 ± 0.01

**Table 4:** Multiple linear regression results on outcome variable DNA damage (OTM value).

Variable	Estimate	95% CI	*p*-Value
Intercept	33.65	32.20 to 35.10	<0.0001
Gender ^a^ (Female)	0.32	−1.12 to 1.77	0.66
Age ^b^ (Old)	4.63	3.18 to 6.07	<0.0001
DEP ^c^ (Exposure)	13.84	12.39 to 15.28	<0.0001

^a^ Reference group: Male. ^b^ Reference group: Young. ^c^ Reference group: Unexposed.

## Data Availability

Not applicable.
